# Development and validation of a questionnaire to assess healthcare personnel competence in cardiac arrest and resuscitation in pregnancy

**DOI:** 10.1371/journal.pone.0232984

**Published:** 2020-05-12

**Authors:** Ann-Chatrin L. Leonardsen, Edel J. Svendsen, Grethe B. Heitmann, Adam Dhayyat, Ann Morris, Katrine D. Sjøborg, Richard M. Olsen, Camilla Hardeland

**Affiliations:** 1 Department of Health and Welfare, Ostfold University College, Halden, Norway; 2 Department of Anesthesiology, Ostfold Hospital Trust, Grålum, Norway; 3 Department of Health and Society, University of Oslo, Oslo, Norway; 4 Department of Medicine, Ostfold Hospital Trust, Grålum, Norway; 5 Department of Obstetrics and Gynecology, Ostfold Hospital Trust, Grålum, Norway; 6 Department of Competence Development, Ostfold Hospital Trust, Grålum, Norway; Murcia University, Spain, SPAIN

## Abstract

**Background:**

Cardiac arrest is rare in pregnancy, and up-to date competence can be difficult to assess and maintain. The objective of this study was to develop and validate a questionnaire to assess healthcare personnel experiences, self-assessed competence and perception of role and resposibility related to cardiac arrest and cardio-pulmonary resuscitation (CPR) in pregnancy

**Methods:**

The study had a cross-sectional design, developing and validating a questionnaire: the Competence in cardiac arrest and CPR in pregnancy (ComCA-P). Development and validation of the ComCA-P was conducted in three stages: 1) Literature review and expert group panel inputs, 2) a pilot study and 3) a cross-sectional questionnaire study. In stage one, the ComCA-P was developed over several iterations between the researchers, including inputs from an expert group panel consisting of highly competent professionals (n = 11). In stage two, the questionnaire was piloted in a group of healthcare personnel with relevant competence (n = 16). The ComCA-P was then used in a baseline study including healthcare personnel potentially involved in CPR in pregnancy (n = 527) in six hospital wards. Based on these data, internal consistency, intra-class correlations, and confirmatory factor analysis were utilized to validate the questionnaire.

**Results:**

The expert group and pilot study participants evaluated the appropriateness, relevance and accuracy to be high. Formulation of the items was considered appropriate, with no difficulties identified related to content- or face validity. Cronbach’s alpha was 0.8 on the thematic area self-assessment, and 0.73 on the theoretical knowledge area of the ComCA-P. On both the self-assessed competence items and the teoretical knowledge items, Kaiser-Meyer-Olkin was 0.8. Moreover, the Bertletts’ test of sphericity was greater than the critical value for chi-square, and significant (p < .0001).

**Conclusions:**

Findings indicate that the ComCA-P is a valid questionnaire that can be used to assess healthcare personnel competence in cardiac arrest and resuscitation in pregnancy.

## Introduction

In Norway, cardiac arrest and deaths in pregnancy is a rare incident, and the maternal mortality rate in 2015 was 5 in 100,000 pregnancies [1]. Internationally, the prevalence of cardiac arrest in pregnant women varies from 1 in 20,000 to 1 in 50,000 ongoing pregnancies [2, 3]. Treatment of cardiac arrest in pregnant women include provision of cardiopulmonary resuscitation (CPR) and advanced medical treatment. CPR in pregnant women differs from standard CPR, and highly competent personnel is required to ensure optimal outcome [4–6].

At 20 weeks pregnancy (or when the uterus reach umbilicus level), the uterus can lead to aortacaval compression syndrome in patients lying on their back, which results in decreased venous return to the heart. This subsequently limits blood flow to the placenta, and may result in increased morbidity and mortality in both mother and fetus [7]. Chest compression on pregnant women is also made difficult by flared ribs, raised diaphragm, obesity, and breast hypertrophy [4, 8]. Consequently, early perimortem cesarean section (PMCS) is recommended to decrease compression on the venous system, and to improve the probability of return of spontaneous circulation (ROSC). The primary purpose of PMCS is to improve the chances of the mother’s survival, and should be considered in cases of maternal cardiac arrest after 20 weeks gestation, irrespective of fetal condition. Emptying the uterus may lead to ROSC, and increase survival rate of the fetus [9–11], dependent on where the cardiac arrest occur [12, 13]. Brain damages due to anoxia occurs at an earlier stage in pregnant women, hence PMCS within 4–5 minutes is an acknowledged practice [13]. In 2015, the American Heart Association released their first scientific statement on guidelines for management of cardiac arrest in pregnancy, and recently consensus has been reached on appropriate resuscitation of a pregnant woman [14].

In standard CPR, resuscitation teams often deviate from algorithms of CPR, and evidence suggests that in addition to technical skills of individual rescuers, human factors such as teamwork affect the quality of CPR [15]. Correct and timely interventions has been shown to affect outcomes after cardiac arrest in pregnancy [16]. This requires highly skilled and competent rescuers. Competence has been described as a combination of knowledge, fitness, assessments and attitudes [17, 18]. The importance of competence in ensuring patient safety and quality has been highlighted in several studies [19–22]. Self-assessment of competence has been linked to healthcare quality and patient safety [19, 22]. Only a few studies have assessed healthcare personnel ability to perform CPR in pregnancy, indicating a need for more education and practice on this area [23–25]. Both Einav et al. [24] and Cohen et al. [25] utilized questionnaires to assess competence in CPR in pregnancy. However, these questionnaires were not validated, included small samples (n = 30/75), and did not include items to assess healthcare personnel perception of their own role and responsibility in such settings.

Self-assessment of knowledge, practical skills and teamwork allow for an awareness of own competence and a possibility to provide tailored training and education of healthcare personnel. Consequently, the objective of this study was to develop and validate a questionnaire to be able to assess healthcare personnel experiences, self-assessed competence and perception of role and responsibilty related to cardiac arrest, CPR and PMCS in pregnant women.

## Materials and methods

### Design

The questionnaire was developed and validated in three stages: (1) Development of a preliminary version of the questionnaire based on literature findings and international guidelines, as well as expert group panel inputs, (2) a pilot study and (3) validation of the questionnaire through baseline data.

### Development of the questionnaire

A sketch of 37 items in the ComCA-P was developed by the researchers based on earlier guidelines and research, as well as the earlier non-validated questionnaires:

Fourteen of the items in the ComCA-P were developed based on contents and findings from two previously used questionnaires:

1. The Einav questionnaire, which include field of expertise and resuscitation experience, a single case vignette of maternal cardiac arrest, followed by nine questions to examine knowledge of existing recommendations for maternal cardiopulmonary resuscitation [24], and
2. The Cohen questionnaire, which in addition included theoretical questions about physiological changes in pregnancy/warning routines/guidelines/positioning/CPR and PMCS. Cohen focused on knowledge deficiencies in four critical areas: need for left uterine displacement, advanced cardiac life support algorithms, physiologic changes of pregnancy, and the recommendation to perform cesarean delivery in parturients (>20 weeks gestation) within 4–5 min of unsuccessful resuscitation for cardiac arrest [25].

In addition, we included six items from the international guideline for treatment of cardiac arrest in pregnancy [14].

Studies have shown a correlation between age, length of experience, education and levels of self-assessed competence [19, 26]. Hence, the demographics area included six items about professional background, work experience, as well as experience with cardiac arrest in pregnancy. Due to anonymity challenges when combining education, experience and age in limited environments, we did not include age as an item. The research group included one of each respectively: nurse anesthetist/CPR coordinator, nurse anesthetist/PhD, pediatric nurse/PhD, registered nurse with a PhD in prehospital cardiac arrest, midwife, specialist in internal medicine, obstetrician/PhD and anesthesiologist with specialization in obstetric anesthesia.

### Expert group panel

We used recommendations from the Delphi technique to develop a self-report questionnaire: the ComCA-P (Competence in Cardiac Arrest in Pregnancy). The Delphi technique is suitable to obtain expert opinions in a systematic manner, and includes four steps: 1) expert input, 2) interaction with feedback, 3) statistical group responses, and 4) confidentiality [27, 28]. In the current study, we included the first two steps. In-line with Keeney et al. [28], and Bing-Jonsson et al. [29], we defined experts as ‘informed individuals’, ‘specialists in their field’ and ‘people who are knowledgeable with regard to cardiac arrest and resuscitation in pregnancy’. Consequently, the expert group consisted of seven anesthesiologists, three obstetricians and one midwife, knowledgeable in the field of obstetrics and resuscitation, and recommended by other experts (Male, n = 6).

The expert group participated in the development of questions that were not included from the Einav and Cohen questionnaires or the guidelines, and gave several constructive inputs on clarity, wording, and contents of the whole questionnaire, as suggested by Streiner & Norman [30]. The expert group received revisions according to their inputs continously, and all received the questionnaire at least twice. In cases of disagreement we also reviewed the preliminary national guideline in CPR in pregnancy, which is about to be introduced in Norway. E.g. there were discussions related to when a fetus is considered viable, when to perform a PMCS, and when to contact a pediatrician. In addition, answering alternatives were adjusted, and the alternative ‘undecided’ added. Through several email rounds in the expert group, as well as discussions in the research group, consensus was reached, and questions or answering alternatives adjusted accordingly.

### Pilot study

After the development process, the ComCA-P was piloted in ten physicians (anesthesiology/medicine) as well as six midvives, to control for face- and content validity; whether questions were logical and relevant and not leading, wording clear, or if questions could be misinterpreted, as well as the time spent to finish the questionnaire.

### Validation study

In a larger intervention study we plan to compare baseline data and post-intervention data using the ComCA-P. The intervention consists of different competence-improving initiatives, such as simulation, table-top discussions and an electronical learning program. Baseline data were used to validate the ComCA-P. The study was conducted in six different hospital departments; maternity, anesthesiology, intensive care, emergency, medical surveillance, and a postoperative anesthesia care unit (PACU). A purposive sampling method, identifying and selecting individuals that are proficient and well-informed with the phenomenon of interest, was used: All healthcare personnel potentially involved in resuscitation in the six departments respectively, were invited to participate (n = 527). This included medical physicians, anesthesiologists, pediatricians, gynaecologists, obstetricians, midwives, nurses, nurses with a specialization (anesthesia, critical care, emergency care), and childrens assistant nurses. Inclusion criteria waspersonnel with 50% clinical work or more. The paper-based questionnaire was distributed to all healthcare personnel in each ward by study- nurses, who also did follow-ups on non-responders. Completed questionnaires were returned in sealed boxes in a separate room at each location. Data were collected over three weeks in March 2019.

### Ethics approval and consent to participate

The study was based on the principles stated in the Declaration of Helsinki; on anonymity, voluntary, informed consent and the right to withdraw without any negative consequences [31]. Participants in the expert group panel and the pilot study were initially contacted via e-mail with information about the project and an invitation to participate in an expert group or the pilot study respectively. Information in all stageswere treated confidential. In the baseline study, a returned, completed questionnaire was assumed as consent to participate. Here, participants had no opportunity to withdraw, since data were unidentifiable. Participants received information about the purpose and nature of the study, potential benefits and risks and that it would not be possible to recognize them in presentation of results. Data were kept in the research area of a safe, internal zone (password and user access) at the university college.

In Norway, the Regional Committees for Medical and Health Research Ethics (REC) are responsible for approving medical and health related research projects. When patient data is not involved in the project, we do not need approval from REC to perform the study. However, approval from the Norwegian Center for Research Data (NSD) was obtained to be able to include healthcare personnel (reference number 558373).

### Statistical analysis

Data from the baseline study were analyzed using the Statistical Package for the Social Sciences (SPSS), version 25. Internal consistency (reliability) of the ComCA-P was measured using Cronbach’s alpha [32, 33]. In addition, we conducted a confirmatory factor analysis; Kaiser-Meyer-Olkin (KMO) and Bartletts’ test of spericity, to test sampling adequacy and variance among variables [34, 35].

Descriptive statistics and frequenzies were used to present characteristics of the sample. Since data were not normally distributed, the continous variables are displayed as median, mean and standard deviation. P values < 0.05 were considered statistically significant.

## Results

### Questionnaire items

The questionnaire consists of 37 items distributed in the five areas as follows: 1) demographics; six items 2) courses and training; four items 3) self-assessed competence; eight items, 4) roles/responsibility; three items, and 5) theoretical knowledge about CPR and PMCS; 16 items.

Demographic data on professional background, work experience, and experience with cardiac arrest in pregnancy were included. In the area ‘courses and training’ two items regarding participation in courses in CPR within the last year (yes/no) and type of course (basic, advanced, other) were included. Moreover, two items about perceived need for more education and training were included (strongly disagree/disagree/neither agree or disagree/agree/strongly agree)). In the ‘self-assessed competence’ area, eight items from the new guideline were included; warning routines, positioning, airway-handling, drug administration before and after delivery, PMCS, defibrillation routines and general competence.

In the area ‘roles/responsibility’, we included three items about the participants’ role and responsibility in CPR in pregnancy- situations (Do you know what your role and responsibility is in CPR in a pregnant woman? What is the content of this role? Do you have any thoughts about what this role could include? Free-text answers).

Finally, sixteen theoretical items about physiological changes in pregnancy/warning routines/guidelines/positioning/CPR (6), and PMCS (10) were included, with pre-defined answering alternatives. The ComCA-P was originally developed in Norwegian. Table 1 gives an overview of the ComCA-P items.

**Table 1:** The items in the ComCA-P, including response alternatives, translated to English.

Courses and training (n = 4)	Self-assessed competence (n = 8)	Roles/responsibility (n = 3)	Theoretical knowledge (n = 16)
Have you participated in CPR courses during the last year? (Yes/No) If yes, what sort of course? (Advanced CPR/CPR for Healthcare personnel/Other) How would you assess your need for more education in CPR in pregnant women with cardiac arrest?* How would you assess your need for more training in CPR in pregnant women with cardiac arrest?*	I have competence in warning routines* I have competence in positioning* I have competence in airway handling* I have competence about drug administration before delivery* I have competence about drug administration after delivery* I have competence about routines for perimortem cesarean section* I have competence about routines for defibrillation* My competence in CPR in pregnancy is overall^&^	Are you familiar with your own role/responisbility in the team in CPR in pregnancy? (Yes/No) If yes, what do this role include? (free-text) If no, what would you think this role should include? (free-text)	From what gestation week will the uterus probably affect the circulation in patients on their back? (0-12/ 13-19/ 20+/ 32+/ never) In what relation should compressions/ respirations be conducted? (30:2/15:1) What position is optimal during CPR? (back/uterus pulled to the mothers’ left side/30 degree left) The procedure should start within….. minutes after cardiac arrest (2/4/6/10) The baby should be delivered within…..minutes after start of procedure (1/3/5/7) Automated compression machine (LUCAS) should not be used until the baby has been delivered** Vaginal delivery should be preferred to PMCS in women with full opening** PMCS is performed when the uterus is at umilical level** A pediatrician has to be called when the gestational age is above 24 weeks** After ROSC, liberal administration of syntocinon is recommended** Are there local, specific warning routines in CPR in pregnancy?*** Are there national guidelines for CPR in pregnancy?*** What are the considerations related to • intravenous access^¤^ • anesthesia before PMCS^¤^ • other preparations before PMCS^¤^ • equipment needed before PMCS^¤^

*Response alternatives: strongly disagree/disagree/neither agree or disagree/agree/strongly agree.

^&^ very low/low/average/good/very good.

** right/wrong/undecided.

*** yes/no/undecided.

^¤^ = free-text. ‘Undecided’ included in all theoretical items. ROSC = return of spontaneous circulation.

### Face-value and feasibility

The pilot testing revealed only few problematic issues, most related to adding a response alternative for «undecided» or «do not know» when appropriate. Moreover, the self-assessment items that read e.g. «knowledge about positioning…» were revised to «competence in positioning…» after feed-back and discussions. Questions were judged logical and relevant without leading, wording clear, with a low risk of misinterpretation, hence face and content validity was assumed. To ensure construct validity of the questionnaire, it was necessary to ensure that a suitable and appropriate conceptual framework was developed that identified those aspects that were considered to be important and relevant for the specific target group to know. These concepts were identified by the expert group.

Participants reported to use 5–10 minutes for completion of the ComCA-P.

### Validation

A total of 251 participants responded to the baseline questionnaire (47.6%). Responders had a median professional experience after basic education of 16.9 years (SD 11), and 10 years after specialization (SD 9.5). Table 2 gives an overview of responders’ demographics, courses and training (not divided into departments due to confidentiality).

**Table 2:** Descriptives of responders’ demographics (n = 251).

**Department**	Maternity 38.6%
	Anesthesiology 6.4%
	Intensive care 15.1%
	Emergency 16.3%
	Medical surveillance 18.7%
	Postoperative anesthesia care unit 4.8%
**Professional background**	Childrens assistant nurses 8.6%
	Nurse 19.6%
	Specialist nurse 50.2%
	Physician 21.7%
**Specialization**	Anesthesiology 20.1%
	Critical care 26.8%
	Emergency care 5.4%
	Midwife 36.2%
	Obstetrics/gynaecology 6.7%
	Medicine 3.4%
	Other 1.4%
**Years of experience since completed basic education as a nurse/physician**	16.9 (11)
**Years of experience since completed specialization**	12.1(9.5)
**Participated in CPR courses the past year**	71.7%
**Participated in CPR in pregnant women**	10.4%
**Need more education**	Strongly disagree 0.8%
	Disagree 1.2%
	Neither agree or disagree 19.9%
	Agree 41.4%
	Strongly agree 36.7%
**Need more training**	Strongly disagree 0.8%
	Disagree 0.8%
	Neither agree or disagree 16.4%
	Agree 45.2%
	Strongly agree 36.8%

Abbreviations; ‘Childrens’ assistant nurse’ is high school level education (not academic) in Norway. ‘Specialist nurse’ include nurse anesthetists, critical care nurses, emergency care nurses and midwives. ‘Physician’ include both medical doctors as well as anesthesiologists/obstetricians/gynaecologists with or without formal specialization. ‘Other’ = e.g. geriatrics or cardiology. Years of experience, reported in mean, years. Standard deviation in parenthesis. CPR = cardiopulmonary resuscitation. ‘Need more education/training’: percentage answering ‘yes’.

The Cronbach’s alpha was 0.8 on the thematic area self-assessment, and 0.73 on the theoretical knowledge area of the ComCA-P. Hence internal consistency was good to acceptable [32, 33]. On both the self-assessed competence items and the teoretical knowledge items, Kaiser-Meyer-Olkin was 0.8, which is assumed a meritorous [34, 35]. Moreover, the Bertletts’ test of sphericity was greater than the critical value for chi-square, and significant (p < .0001). Hence, we assume that there is unlikely to be a problem with multicollinearity, and that there are sufficient items for each thematic area. Table 3 gives an overview of items included in the different analyses.

**Table 3:** Items included in the analyses.

Chronbach’s alpha	Kaiser-Meyer-Olkin/Bertletts’ test of sphericity
‘Self-assessed competence area’, including eight items; warning routines, positioning, airway-handling, drug administration before and after delivery, PMCS, defibrillation routines and general competence.	‘Self-assessed competence area’, including eight items; warning routines, positioning, airway-handling, drug administration before and after delivery, PMCS, defibrillation routines and general competence.
‘Theoretical knowledge area’, including 12 items about physiological changes in pregnancy/warning routines/guidelines/positioning/CPR and PMCS (not included: items with free-text answers, see Table 1)	‘Theoretical knowledge area’, including 12 items about physiological changes in pregnancy/warning routines/guidelines/positioning/CPR and PMCS (not included: items with free-text answers, see Table 1)

## Discussion

Resuscitation in pregnancy is complex due to several factors unique to pregnancy: the altered physiological state, the requirement to consider both maternal and fetal issues during resuscitation, and the consequent possibility of PMCS. Hence, competence is essential to ensure optimal outcome. Consequently, healthcare institutions need access to tailored assessment instruments that can help monitor competence and areas herein that need improvement. In this study, we developed and validated a questionnaire- the Competence in Cardiac Arrest in Pregnancy (ComCA-P), which is shown as a feasible tool to assess healthcare personnel competence regarding cardiac arrest and resuscitation in pregnant women.

The expert group gave several constructive inputs on clarity, wording, and contents, as suggested by Streiner & Norman [30]. The experts heterogeneity allowed for a range of views to be explored, and their expertise in the field and commitment to the study are additional signs of content validity [29]. Consensus is an essential part of content validity, because it signifies acceptability and recognizability of the questionnaire content to relevant personnel [27]. The identified aspects that were considered important and relevant for the experts, as well as for the participants in the pilot study, were supported by those included by Einav et al. [24] and Cohen et al. [25]. Even though these questionnaires were never validated, and the samples only 30 and 75 respectively, they were developed by and completed within groups of healthcare personnel with competence in CPR and in pregnancy.

Earlier studies show that healthcare personnel are divided in their opinions regarding every choice of action in resuscitation in pregnancy: positioning, airway handling, compressions, medications and PMCS [12, 24, 36]. In addition, emerging evidence suggests that human factors such as teamwork and leadership affect adherence to algorithms and hence the outcome of CPR [15]. Hence, assessment of competence in personnel as well as assessment of teamwork is essential when aiming to provide optimal treatment for pregnant cardiac arrest patients. The ComCA-P include an assessment of personnel perspectives of role and responsibility in CPR settings, which the pre-existing questionnaires did not. Conclusively, the ComCA-P is the first validated tool to make this assessment. In addition, results from the ComCA-P assessment contribute insights into knowledge and competence gaps that need focus during the planning and implementation of improvement initiatives regarding resuscitation in pregnant women; a study from the USA indicated that median time for starting CPR decreased under 1 minute after introducing a structured educational program [37]. Successful implementation is a function of knowledge, context and fascilitation [38].

Knowledge gaps have been shown significant in the science of maternal resuscitation [8]. Hence, the current study fills this gap by adding a validated tool for assessment of competence in cardiac arrest and CPR in pregnancy. Moreover, we suggest that the ComCA-P may easily be adjusted for other circumstances, e.g in cardiac arrest and resuscitation in intensive care units (ComCA-ICU), trauma, anaphylaxis, or morbid obesity [39].

## Limitations

Self-assessment is subjective and based on individual interpretation of the concept of competence. Studies have reported varying degrees of agreement between self-perceived and objectively measured competence [40, 41]. Nevertheless, self-assessment measures is widely utilized and accepted.

Development of the items in the questionnaire was based on non-validated questionnaires used in small samples. The validity of the study could also be diminished by not carrying out a study on the temporal stability of the questionnaire. Nevertheless, both these questionnaires were used in expert-settings, consisting of specialists in anesthesiology, obstetrics and resuscitation. In addition, we included items from the consensus-based international guidelines. Due to the transparent methodology, we claim that the ComCA-P include relevant, valid and thrustworthy items that measures what it is intended to measure (content validity).

The baseline study was conducted in one hospital only, which may limit the external validity (reliability) and generalizability of the study. Nevertheless, six different wards as well as healthcare personnel with different professional background and experience were included, and we achieved significant answers to several of the thematic areas.

Moreover participants had the opportunity to discuss with colleagues before responding to the theoretical questions. Practical tests, observation of simulated cases or theoretical exams may give a more «true» overview of the participants’ acutal competence.

## Conclusion

The ComCA-P is a valid questionnaire that can be used to assess healthcare personnel competence in cardiac arrest and resuscitation in pregnancy, as well as in perimortem cesearen section. Moreover, the ComCA-P can be used to assess discrepancies in attitudes to role and responsibility within CPR teams. This knowledge is essential when focusing on education, training and quality improvement initiatives related to this rare incident. In addition, the ComCA-P may be adjusted to other conditions that deviate from regular CPR (or even as an assessment of competence in resuscitation, the ComCA-R).

## Supporting information

S1 File The questionnaire. (DOCX)

S1 Data Baseline data. (SAV)
