# Supplementary material for: Development and validation of a questionnaire to assess healthcare personnel competence in cardiac arrest and resuscitation in pregnancy
Source: PLoS One. 2020 May 12;15(5):e0232984. doi: 10.1371/journal.pone.0232984 (PMC7217426; doi:10.1371/journal.pone.0232984)
Supplement: S1 File — (DOCX) [file pone.0232984.s001.docx]

**RESUSCITATION OF PREGNANT WOMEN WITH CARDIAC ARREST**

# Background

## What is your professional background?

Nurse

Nurse with further education

Type of further education (write):

Other education(write):

Doctor

Specialist

Speciality (write):

**How many years since you ended basic education as a nurse/doctor? Write:**

_____________ years

**How many years since you ended your further education/specialization? Write:**

**______________**years

**Courses**

## Have you participated in courses in resuscitation during the last year?

| Yes | No |
| --- | --- |

**If yes, what kind of course? (several possible answers)**

ACPR BCPR Other Undecided

(ACPR: advanced cardio pulmonary resuscitation, BCPR: basic cardio pulmonary resuscitation).

# Experience with resuscitation in pregnancy

## Have you ever participated in resuscitation in a pregnant woman with cardiac arrest?

| Yes | No |
| --- | --- |

**If yes, when was this (apply approximate time):**

**____________________________________________**

**Knowledge and competence**

**How would you define your own need for more education about resuscitation in pregnant women with cardiac arrest?**

1=very low 2=low 3=average 4= high 5= very high

## 1 2 3 4 5

**How would you define your own need for more training/simulation about resuscitation in pregnant women with cardiac arrest?**

1=very low 2=low 3=average 4= high 5= very high

## 1 2 3 4 5

**Look at the statements below, and self-assess your competence related to these:**

| **Statement** | **Self-assessed competence**  1=Very low, 2=Low, 3=Average, 4=High, 5=Very high | | | | | **Un-decided** |
| --- | --- | --- | --- | --- | --- | --- |
| I have competence in warning routines | **1** | **2** | **3** | **4** | **5** |  |
| I have competence about positioning | **1** | **2** | **3** | **4** | **5** |  |
| I have competence about airway handeling | **1** | **2** | **3** | **4** | **5** |  |
| I have competence about medication **before** the baby is delivered | **1** | **2** | **3** | **4** | **5** |  |
| I have competence about medication **after** the baby is delivered | **1** | **2** | **3** | **4** | **5** |  |
| I have competence about routines for perimortem caesarean section | **1** | **2** | **3** | **4** | **5** |  |
| I have competence about routines for defibrillation | **1** | **2** | **3** | **4** | **5** |  |
| My overall competence in cardio pulmonary resuscitation in pregnancy is | **1** | **2** | **3** | **4** | **5** |  |

**From which week of pregnancy will the size of the uterus affect circulation in a women lying on her back?**

| 0-12 weeks | 13-19 weeks | 20+ weeks | 32+ weeks | Not at all |
| --- | --- | --- | --- | --- |
| □ | □ | □ | □ | □ |

## Undecided

□

## Are there local warning routines for cardiac arrest in pregnancy at your hospital?

| Yes | No |
| --- | --- |
| □ | □ |
| **Undecided** |  |

## 

## Are there national guidelines for cardiac arrest in pregnancy?

| Yes | No |
| --- | --- |
| □ | □ |
| **Undecided**  □ |  |

## In what relation do you perform compressions and breathing in pregnant women with cardiac arrest?

| 30:2 | 15:1 |
| --- | --- |
| □ | □ |

## Undecided

##

## □

**What is the correct position during resuscitation in pregnant women with cardiac arrest?**

On the back Uterus is dragged to the left side 30 degrees to the left (pillow)

□ □ □

## Udecided

## □

**What considerations do you have regarding intravenous access? (write with your own words)**

**Role and function**

**Do you know which role/function you have in the resuscitation team during resuscitation in a pregnant woman with cardiac arrest?**

| Yes | No |
| --- | --- |
| □ | □ |

## Undecided

## □

1. **If yes, what is this role/function (write short, with your own words)**

## If no/undecided, what is your opinion of what this role/function should be? (write short, with your own words)

#

# Knowledge about perimortem caesarean section

## What are the time limits for perimortem caesarean section?

The procedure should start within…….minutes after the cardiac arrest has occured (upper limit):

2 4 6 10

**Undecided** □

The baby should be delivered within…….minutes after the procedure was initiated

1 3 5 7

**Undecided**  □

## Describe shortly, with your own words essential considerations regarding anesthesia/preparations/equipment before and during perimortem caesarean section

Anesthesia:

Preparations:

Equipment:____________________________________________________________________

**Undecided**  □

**Assesss whether these statements are correct or uncorrect:**

LUCAS (automatic heart compression machine) is not to be used before the baby is delivered (intrahospital)

Correct Uncorrect

 

**Undecided**  □

Vaginal delivery is preferred above perimortem caesarean section in women with full opening

Correct Uncorrect

 

**Undecided**  □

Perimortem caesarean section is performed when the uterus reaches umibilicus level

Correct Uncorrect

 

**Undecided**  □

A pediatrician is contacted at gestational age above 24 weeks

Correct Uncorrect

 

**Undecided**  □

When spontaneous circulation has returned liberal administration of oksytocin (Syntocinon) is recommended to stop the bleeding

Correct Uncorrect

 

**Undecided**  □
